# Cardiovascular System Response to Carbon Dioxide and Exercise in Oxygen-Enriched Environment at 3800 m

**DOI:** 10.3390/ijerph120911781

**Published:** 2015-09-18

**Authors:** Guohui Liu, Xiaopeng Liu, Zhifeng Qin, Zhao Gu, Guiyou Wang, Weiru Shi, Dongqing Wen, Lihua Yu, Yongchang Luo, Huajun Xiao

**Affiliations:** 1 School of Aeronautic Science and Engineering, Beihang University, Beijing 100191, China; E-Mail: guohui_l@buaa.edu.cn; 2 High Altitude Physiology Laboratory, Institute of Aviation Medicine, Air Force, Beijing 100142, China; E-Mails: liu_xiao_peng@hotmail.com (X.L.); zhifeng_q@hotmail.com (Z.Q.); guzhao_gz@hotmail.com (Z.G.); guiyouw@hotmail.com (G.W.); swr7823@sohu.com (W.S.); wendongqing@gmail.com (D.W.); lihua19780420@163.com (L.Y.)

**Keywords:** heart rate variability, heart rate, blood pressure, cardiac autonomic modulation, exercise

## Abstract

*Background:* This study explores the responses of the cardiovascular system as humans exercise in an oxygen-enriched room at high altitude under various concentrations of CO_2_. *Methods:* The study utilized a hypobaric chamber set to the following specifications: 3800 m altitude with 25% O_2_ and different CO_2_ concentrations of 0.5% (C1), 3.0% (C2) and 5.0% (C3). Subjects exercised for 3 min three times, separated by 30 min resting periods in the above-mentioned conditions, at sea level (SL) and at 3800 m altitude (HA). The changes of heart rate variability, heart rate and blood pressure were analyzed. *Results:* Total power (TP) and high frequency power (HF) decreased notably during post-exercise at HA. HF increased prominently earlier the post-exercise period at 3800 m altitude with 25% O_2_ and 5.0% CO_2_ (C3), while low frequency power (LF) changed barely in all tests. The ratios of LF/HF were significantly higher during post-exercise in HA, and lower after high intensity exercise in C3. Heart rate and systolic blood pressure increased significantly in HA and C3. *Conclusions:* Parasympathetic activity dominated in cardiac autonomic modulation, and heart rate and blood pressure increased significantly after high intensity exercise in C3.

## 1. Introduction

It is well known that low oxygen partial pressure (${\text{P}}_{{\text{O}}_{2}}$) that is inherent in high altitude atmosphere can cause physiological reactions in the cardiovascular system, nervous system and musculoskeletal system, *etc.* [1,2,3,4,5,6,7,8]. Some researchers reported that lowlanders who have sojourned into high altitudes would take seven months or more to acclimatize to hypoxia of high altitude areas [9,10]. However, oxygen-enriched environments offer an effective way to alleviate the deleterious effect of hypoxia [11,12,13,14]. Studies have focused on comparing the oxygen concentrations of oxygen-enriched room in equivalent altitudes, identifying a safe limit of the oxygen concentration, and calculating and measuring indoor CO_2_ concentration [14,15,16,17]. Increasing O_2_ input and/or reducing ventilation are conventional methods for constructing an oxygen-enriched environment. However, more carbon dioxide (CO_2_) gas would be exhaled into this relatively confined space, resulting in an elevation of CO_2_ concentration when humans undertook heavy activities in an oxygen-enriched room. CO_2_ is considered the first pollutant of indoor air quality (IAQ), which would trigger physiological reactions [18]. In addition, several studies have demonstrated that the CO_2_ concentration surpasses the ASHRAE (American Society of Heating, Refrigerating, and Air Conditioning Engineers) standard in normal buildings [19,20,21,22]. Furthermore, it was observed that high concentration CO_2_ gas in some oxygen-enriched buildings that are located in Tibet, China.

In psychiatry, CO_2_ inhalation has often been applied and is a well-established experimental model of human panic. The panic symptoms could be triggered to different extents, depending on the concentration of CO_2_ used. CO_2_ concentrations from 4% to 65% and inhalation duration from 15 s to 20 min were used in these studies [23,24]. These studies showed that parasympathetic activity would dominate in cardiac autonomic modulation (CAM), when the concentration of CO_2_ accumulated in the human body is high enough to excite the baroreceptor. In pathology, for obtaining hypercapnia, hypercapnic gas that contains high concentrations of CO_2_ gas with/without normobaric hypoxia was applied to subjects during exercise or resting [25,26,27]. In this study, CO_2_ was identified as an air pollutant in the oxygen-enriched room at high altitude. The positive role of oxygen-enrichment at high altitude was clarified. Meanwhile the effects of high CO_2_ concentration and hypobaric hypoxia were neglected. This study focused on how the responses of cardiovascular system to CO_2_ with concentration just higher than the standards of indoor air quality, as humans undertook activities in the oxygen-enriched room and breathed this polluted air for a long duration. Moreover the environments actually contained moderate hypoxia and different levels of high CO_2_ concentration. During acute exposure to the moderate hypoxia and high CO_2_ inhalation, the present study hypothesized that the latter would be a main factor that influenced the cardiovascular system. Additionally, whether or not there exists a significant change in the cardiovascular system was related to the concentrations and inhalation duration of CO_2_ gas.

## 2. Methodology and Measurements

### 2.1. Characteristics of the Study Subjects

Six male subjects, all nonsmokers, living at sea level, volunteered to participate in this study. They were informed of the testing procedures as well as the risks and benefits of the investigation. By filling out a health history questionnaire, participants were free of the following conditions: hypertension, cardiovascular and pulmonary disease, orthopedic limitations to exercise, and using medications of β-blocker or β-agonist. Informed consent was obtained from all individual participants included in the study. The characteristics of subjects were measured at sea level as shown in Table 1.

**Table 1:** Characteristics of subjects.

Subject	Age (years)	Height (cm)	Weight (kg)	HR (bmp)	SBP (mmHg)	DBP (mmHg)
1#	24	173	64	64	112	72
2#	26	175	66	67	116	78
3#	22	179	62	62	109	68
4#	25	170	63	69	110	76
5#	25	181	69	61	103	74
6#	28	172	65	66	114	69
Mean ± SD	25.0 ± 2.0	175.0 ± 4.2	64.8 ± 2.5	64.8 ± 3.1	110.7 ± 4.6	72.8 ± 3.9

HR, heart rate. SBP, systolic blood pressure; DBP, diastolic blood pressure.

### 2.2. Experimental Conditions

In the pre-experiment, mixture gas was supplied to subjects through mask or sealed helmet first. The CO_2_ and O_2_ concentrations of mixture gas satisfied the requirement of inspired air. However, due to the small space of mask or sealed helmet, it was found that the CO_2_ concentration fluctuated strongly during exercise tests. Additionally, subjects reported that expiratory resistance of mask is an external load. Therefore, pure O_2_ and CO_2_ gases (both at least 99.50% pure) were released into the 5.0 m (length) × 1.9 m (width) × 2.0 m (height) hypobaric chamber at the same time whereby internal pressure was equivalent to 3800 m. Through calculating and controlling the ventilation rate of the hypobaric chamber and release rate of O_2_ and CO_2_ gases, three steady high concentrations of CO_2_ and oxygen-enrichment environments in hypoxia were reached (Table 2). The O_2_ and CO_2_ concentrations were monitored by a process mass spectrometer (MGA 1200 EC™, US), and the temperature was kept at 23–25 °C for all tests.

The oxygen enriched environment aims to reduce the equivalent altitude by improving partial pressure of inspired oxygen (PIO_2_). Every 1% increase in oxygen concentration reduces the equivalent altitude by about 300 m [1,14,17]. In this study, as we increased the O_2_ concentration by 4% from 21% to 25% at the altitude of 3800 m (HA), the real PIO_2_ was equal to about 2600 m (PIO_2_ ≈ 118.6 Torr, C1, C2 and C3), which was identified as moderate hypoxia [28]. In addition, 0.5%, 3.0% and 5.0% of CO_2_ concentrations in C1, C2 and C3, were equivalent to 0.31%, 1.89% and 3.14% at sea level, respectively.

**Table 2:** Characteristics of the experimental conditions.

Symbol	Altitude (m)	Barometric Pressure (mmHg)	O_2_ Concentration (%)	CO_2_ Concentration (%)	Experiment Environmental Conditions
SL	0	760.4–763.6	21%	0.03	No operating vacuum pumps and chamber gate open
HA	3800	475.3–477.4	21%	0.03	Simulating 3800 m altitude without releasing O_2_ and CO_2_ gases
C1	3800	476.2–477.8	25%	0.5	Releasing O_2_ and CO_2_ gases into hypobaric chamber that was simulating 3800m, and controlling the ventilation rate of hypobaric chamber and release rate of O_2_ and CO_2_ gases
C2	3800	476.9–478.5	25%	3.0	
C3	3800	476.7–478.2	25%	5.0	

The temperature of measuring barometric pressure was 23 to 25 °C.

### 2.3. Exercise Test Protocol

**Figure 1:**
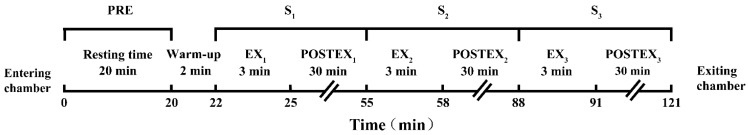
Exercise test protocol. PRE, pre-exercise. S_1_, S_2_ and S_3_, segment 1, segment 2 and segment 3. EX_1_, EX_2_ and EX_3_, the first, second and third exercise. POSTEX_1_, POSTEX_2_ and POSTEX_3_, the first, second and third interval.

Subjects entered the chamber, and rested motionless for 20 min (pre-exercise, PRE). During this period, it took about 3 min to reach the experimental condition requirements. Prior to the exercise test, subjects engaged in a 2 min warm-up of freewheeling on a calibrated cycle ergometer (SCIFIT ISO-1000, USA). Subjects also cycled the cycle ergometer to complete the exercise test, which was composed of the three segment sequences: segment 1 (S_1_), segment 2 (S_2_) and segment 3 (S_3_). Each segment included a 3-min exercise period and a 30-min resting period. During each exercise test, subjects exercised three times (EX_1_, EX_2_ and EX_3_) at the same intensity and rested three times (POSTEX_1_, POSTEX_2_ and POSTEX_3_), alternately (Figure 1). Two cycling intensities were set: low (LI, 450 kg·m·min^−1^ (75 watt, 1.5 kg at 50 rpm)) and high (HI, 900 kg·m·min^−1^ (150 watt, 3.0 kg at 50 rpm)). During the resting period, subjects sat on a chair sedentarily. All exercise tests were conducted between 9 a.m. and 12 a.m. in random order, and were separated by two days. The study was conducted in accordance with the Declaration of Helsinki, and the protocol was approved by the Research and Ethics Committee of Institute of Aviation Medicine, China (JNKT0014/005).

### 2.4. Measurement

From entering to exiting the chamber, the blood pressure (BP) was recorded with a BP cuff (FMS Finometer Pro, Netherlands). The heart rate (HR) was recorded, and the ECG signal was sampled at 1000 Hz by PowerLab (AD Instruments, Castle Hill, Australia), and stored for further analysis. The ECG waveform was re-sampled at 4.0 Hz. The Fast Fourier transform (FFT) method was applied to obtain the spectral components of heart rate variability (HRV). The power spectrum was analyzed for total-(TP; 0.0–0.4 Hz (*ln*(ms^2^))), low-(LF; 0.04–0.15 Hz (*ln*(ms^2^))) and high-(HF; 0.15–0.4 Hz (*ln*(ms^2^))) frequency power. The ratio of LF/HF was calculated to better quantify the balance of sympathetic/parasympathetic action [29]. The ECG recordings of each 30 min resting period were divided into six 5 min sections (0–5, 5–10, 10–15, 15–20, 20–25 and 25–30 min). HRV analysis excluded the initial 5 min (0–5 of POSTEX_1_, POSTEX_2_ and POSTEX_3_) as heart rate changed rapidly at the beginning of post-exercise, and also excluded the final 5 min (25–30 of POSTEX_1_, POSTEX_2_ and POSTEX_3_) because of preparation for the next exercise.

### 2.5. Statistical Analysis

All statistical analyses were done with a statistical software package (SPSS for Windows, 19.0). In HRV analysis, the SL data were identified as a baseline. The reported measure ANOVA was used to compare HA, C1, C2 and C3 to SL at the same segment and exercise intensity. Five (experimental conditions) × 3 (segments) repeated measure ANOVA was used to compare the effects of the experimental condition and/or exercise intensity on heart rate and blood pressure. A *p*-value less than 0.05 was considered statistically significant.

## 3. Results

### 3.1. HRV Analysis

**Figure 2:**
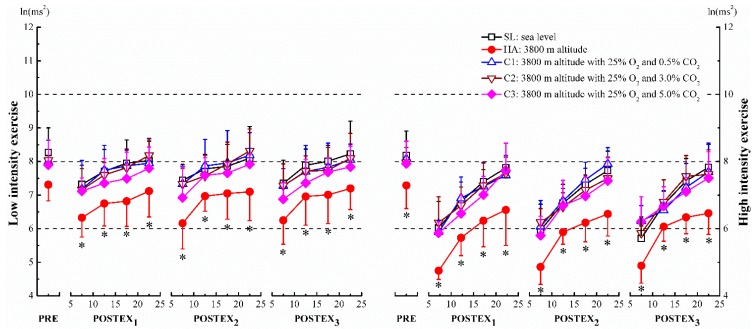
Total power (TP) in pre-exercise (PRE) and post-exercise (POSTEX_1_, POSTEX_2_ and POSTEX_3_). * *p* < 0.05, compared with SL in the same segment and exercise intensity.

**Figure 3:**
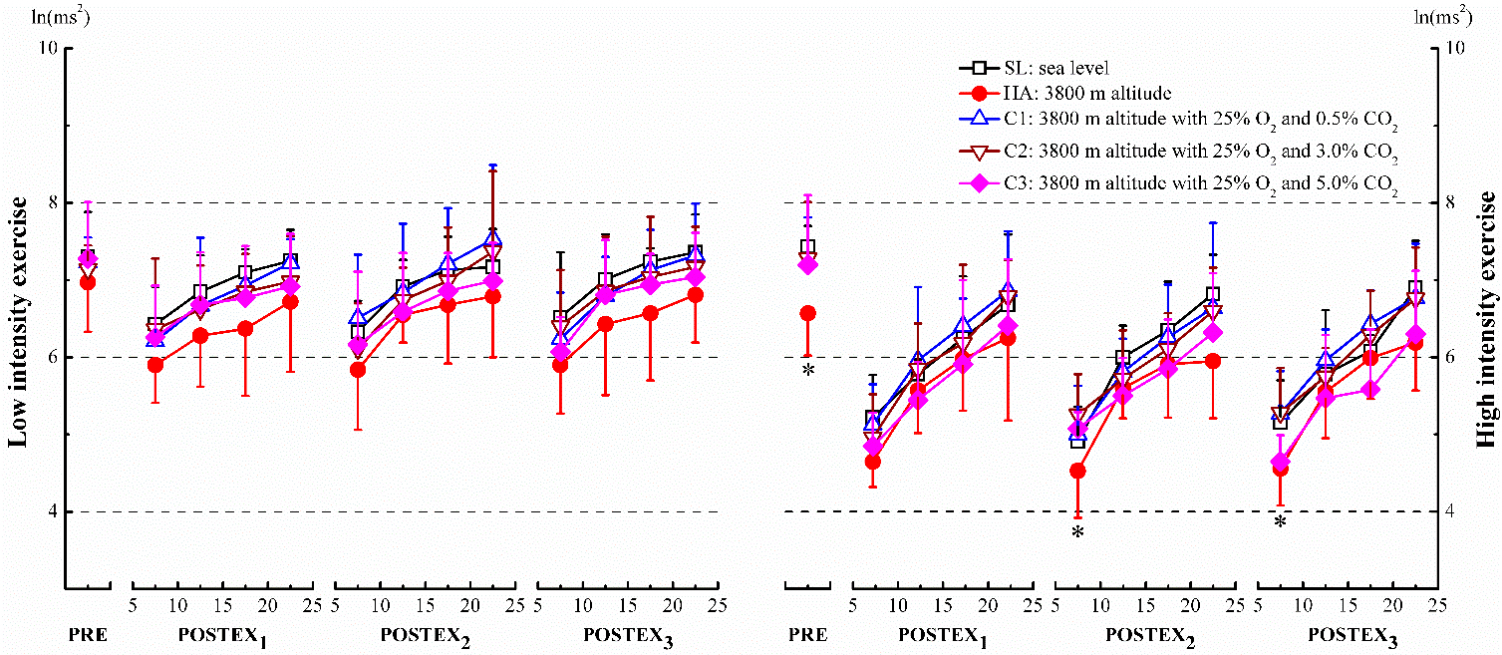
Low frequency (LF) in pre-exercise (PRE) and post-exercise (POSTEX_1_, POSTEX_2_ and POSTEX_3_). * *p* < 0.05, compared with SL in the same segment and exercise intensity.

**Figure 4:**
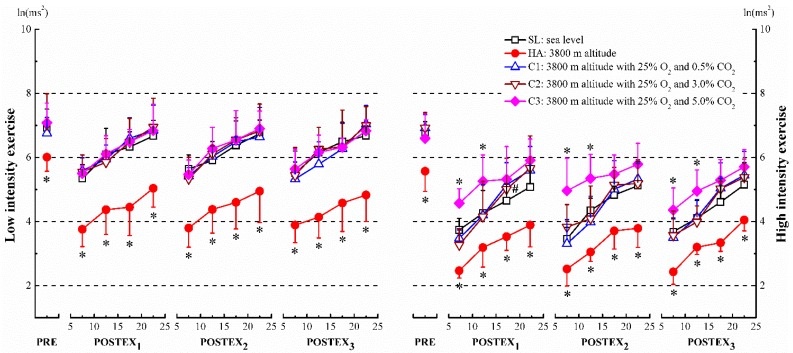
High frequency (HF) in pre-exercise (PRE) and post-exercise (POSTEX_1_, POSTEX_2_ and POSTEX_3_). * *p* < 0.05, compared with SL in the same segment and exercise intensity. # *p* = 0.049, C2 compared with SL in POSTEX_1_ 15–20 after high intensity exercise.

**Figure 5:**
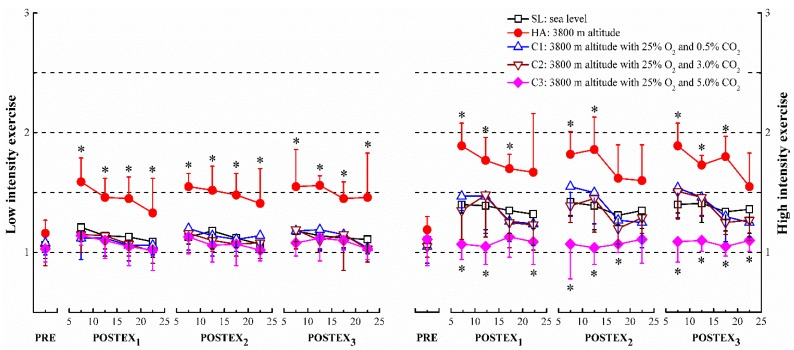
The ratios of low frequency/high frequency (LF/HF) in pre-exercise (PRE) and post-exercise (POSTEX_1_, POSTEX_2_ and POSTEX_3_). * *p* < 0.05, compared with SL in the same segment and exercise intensity.

Compared to the sea level (SL) in the same segment, total power (TP) decreased significantly after the two intensity exercises at high altitude (HA), except for pre-exercise (PRE) before low intensity exercise, where the changes were found to be statistically insignificant (*p* < 0.05, Figure 2). The values of low frequency (LF) were significantly lower than SL after the high intensity exercise, only at pre-exercise, 05–10 min section of post-exercise in segment 2 (POSTEX_2_ 05-10) and segment 3 (POSTEX_3_ 05–10) (*p* < 0.05, Figure 3, right panel). High frequency (HF) was suppressed remarkably during both pre-exercise and post-exercise in comparison with SL (*p* < 0.05, Figure 4). After high intensity exercise at 3800 m altitude with 25% O_2_ and 5.0% CO_2_ (C3), HF at PRE, 05–10, 10–15 sections of each post-exercise (POSTEX_1_, POSTEX_2_ and POSTEX_3_) were significantly higher than those at SL, and the significance was marginal during the 05–10 min section of post-exercise in segment 1 (POSTEX_1_ 05–10) at 3800 m altitude with 25% O_2_ and 3.0% CO_2_ (C2) (*p* < 0.05, Figure 4, right panel). In contrast to SL during post-exercise, the values of low frequency/high frequency (LF/HF) increased significantly in HA, except for 20–25 of POSTEX_1_, 15–20, 20–25 of POSTEX_2_ and 20–25 of POSTEX_3_ after high intensity exercise. The ratios were significantly lower than SL after high intensity exercise in C3 except during 15–20 of POSTEX_1_ and 20-25 of POSTEX_2_ (*p* < 0.05, Figure 5, right panel).

### 3.2. Heart Rate

For the same experimental condition and segment, the maximum values of heart rate during high intensity exercise (HI) were significantly higher than those during low intensity exercise (*p* < 0.05, Table 3). During high and low intensity exercise at 3800 altitude (HA) and 3800 m altitude with 25% O_2_ and 5.0% CO_2_ (C3), maximum values of heart rate during two intensity exercises were significantly higher than those at SL in the same segment (*p* < 0.05, Table 3). This significance did not present in 3800 m altitude with 25% O_2_ and 0.5% CO_2_ (C1) and 3800 m altitude with 25% O_2_ and 3.0% CO_2_ (C2).

During the post-exercise, only the resting heart rate in HA differed significantly from SL (*p* < 0.05, Table 3).

**Table 3:** Heart rate (Mean (SD), bpm) in exercise test.

Exercise Intensity	Sea Level			3800 m Altitude			3800 m Altitude with 25% O_2_ and 0.3% CO_2_			3800 m Altitude with 25% O_2_ and 3.0% CO_2_			3800 m Altitude with 25% O_2_ and 5.0% CO_2_		
	S_1_	S_2_	S_3_	S_1_	S_2_	S_3_	S_1_	S_2_	S_3_	S_1_	S_2_	S_3_	S_1_	S_2_	S_3_
Maximum values of heart rate during exercise															
LI	109.3 (7.8)	105.8 (4.0)	103.5 (1.5)	126.7 (3.7) *	128.3 (3.4) *	130.2 (3.3) *	104.8 (2.1)	107.2 (2.9)	108.2 (4.3)	105.3 (2.7)	108.7 (4.5)	106.7 (4.1)	134.2 (4.2) *	132.8 (3.2) *	137.5 (4.2) *
HI	140.5 (6.5) ^†^	142.3 (9.4) ^†^	138.3 (2.8) ^†^	158.2 (5.3) ^†,^*	161.7 (4.6) ^†,^*	164.0 (6.9) ^†,^*	142.8 (5.7) ^†^	145.0 (6.0) ^†^	141.3 (5.2) ^†^	143.2 (2.5) ^†^	145.8 (3.7) ^†^	142.7 (9.4)	165.3 (6.2) ^†,^*	175.8 (5.6) ^†,^*	177.7 (4.3) ^†,*^
Resting heart rate during post-exercise															
LI	141.7 (4.0)	146.5 (3.3)	143.2 (1.7)	144.5 (5.6)	144.2 (5.8)	148.0 (6.8)	143.2 (5.0)	145.5 (8.1)	147.3 (5.3)	146.3 (6.1)	143.7 (5.7)	142.8 (4.5)	156.7 (6.5) *	158.3 (5.2) *	160.0 (8.7) *
HI	177.7 (6.3) †	183.2 (2.3) ^†^	181.5 (7.2) ^†^	184.8 (6.2) ^†^	179.5 (4.7) ^†^	181.8 (4.2) ^†^	182.3 (2.9) ^†^	183.2 (3.2) ^†^	185.7 (4.5) ^†^	181.7 (4.6) ^†^	182.0 (4.4) ^†^	184.5 (4.9) ^†^	196.3 (6.8) ^†,^*	198.5 (6.9) ^†,^*	201.8 (6.5) ^†,^*

LI, low intensity exercise; HI, high intensity exercise. S_1_, S_2_ and S_3_, segment 1, segment 2 and segment 3. ^†^ *p* < 0.05, significantly different between HI and LI in same experimental condition and segment; * *p* < 0.05, significantly different from SL in the same exercise intensity.

### 3.3. Blood Pressure

The maximum systolic blood pressure (SBP) during high intensity exercise was significantly higher than that during LI in the same experimental condition and segment (*p* < 0.05, Table 4). Compared with sea level in the same segment, maximum SBP increased significantly during low and high intensity exercise in C3 (*p* < 0.05, Table 4). During post-exercise, minimum SBP was not remarkably affected by the exercise intensity or experimental conditions (Table 4). Neither the maximal values nor minimal values of diastolic blood pressure (DBP) changed significantly (Table 5).

**Table 4:** Systolic blood pressure ((Mean (SD), mmHg) in exercise test.

Exercise Intensity	Sea Level			3800 m Altitude			3800 m Altitude with 25% O_2_ and 0.3% CO_2_			3800 m Altitude with 25% O_2_ and 3.0% CO_2_			3800 m Altitude with 25% O_2_ and 5.0% CO_2_		
	S_1_	S_2_	S_3_	S_1_	S_2_	S_3_	S_1_	S_2_	S_3_	S_1_	S_2_	S_3_	S_1_	S_2_	S_3_
Maximum during exercise															
LI	141.7 (4.0)	146.5 (3.3)	143.2 (1.7)	144.5 (5.6)	144.2 (5.8)	148.0 (6.8)	143.2 (5.0)	145.5 (8.1)	147.3 (5.3)	146.3 (6.1)	143.7 (5.7)	142.8 (4.5)	156.7 (6.5) *	158.3 (5.2) *	160.0 (8.7) *
HI	177.7 (6.3) ^†^	183.2 (2.3) ^†^	181.5 (7.2) ^†^	184.8 (6.2) ^†^	179.5 (4.7) ^†^	181.8 (4.2) ^†^	182.3 (2.9) ^†^	183.2 (3.2) ^†^	185.7 (4.5) ^†^	181.7 (4.6) ^†^	182.0 (4.4) ^†^	184.5 (4.9) ^†^	196.3 (6.8) ^†,^*	198.5 (6.9) ^†,^*	201.8 (6.5) ^†,^*
Minimum during post-exercise															
LI	64.3 (2.5)	67.0 (3.9)	65.8 (3.5)	83.5 (3.0) *	82.3 (4.5) *	82.2 (5.9) *	67.7 (4.5)	65.2 (2.9)	68.5 (3.2)	65.5 (3.5)	66.0 (3.2)	69.5 (4.1)	66.8 (2.9)	67.5 (3.0)	69.2 (3.8)
HI	64.0 (5.1)	66.5 (3.1)	68.2 (4.3)	81.2 (4.8) *	80.7 (6.5) *	82.2 (5.9) *	66.2 (4.4)	65.1 (2.9)	69.3 (3.1)	64.2 (3.9)	68.2 (2.7)	69.7 (5.2)	63.7 (3.6)	70.2 (3.6)	61.7 (6.3)

LI, low intensity exercise; HI, high intensity exercise. S_1_, S_2_ and S_3_, segment 1, segment 2 and segment 3. ^†^ *p* < 0.05, significantly different between HI and LI in the same experimental condition and segment; * *p* < 0.05, significantly different with LA in the same exercise intensity.

**Table 5:** Diastolic blood pressure ((Mean (SD), mmHg) in exercise test.

Exercise Intensity	Sea Level			3800 m Altitude			3800 m Altitude with 25% O_2_ and 0.3% CO_2_			3800 m Altitude with 25% O_2_ and 3.0% CO_2_			3800 m Altitude with 25% O_2_ and 5.0% CO_2_		
	S_1_	S_2_	S_3_	S_1_	S_2_	S_3_	S_1_	S_2_	S_3_	S_1_	S_2_	S_3_	S_1_	S_2_	S_3_
Maximum during exercise															
LI	77.3 (4.2)	78.3 (4.2)	72.3 (1.4)	71.7 (1.8)	77.5 (3.8)	75.3 (4.6)	76.3 (5.2)	75.0 (6.0)	69.7 (5.3)	73.7 (4.3)	71.3 (3.9)	77.0 (3.9)	76.7 (2.7)	73.3 (3.6)	77.2 (4.6)
HI	77.2 (2.7)	75.8 (2.4)	71.0 (1.8)	76.2 (4.4)	77.2 (3.7)	73.7 (3.1)	74.3 (3.9)	70.8 (3.9)	76.0 (2.5)	71.2 (3.2)	73.3 (5.5)	76.2 (4.4)	77.0 (3.0)	76.0 (6.0)	72.7 (5.0)
Minimum during post-exercise															
LI	75.0 (2.8)	74.7 (1.8)	71.8 (3.1)	70.5 (4.8)	77.0 (3.7)	75.8 (3.9)	74.7 (2.3)	75.0 (6.0)	72.8 (2.3)	73.0 (2.8)	71.3 (2.1)	76.3 (4.3)	70.7 (4.9)	73.0 (2.2)	77.5 (2.3)
HI	70.0 (4.7)	74.2 (1.9)	73.3 (1.5)	71.0 (2.8)	76.8 (6.0)	74.7 (3.1)	71.8 (2.7)	74.0 (3.8)	74.2 (2.9)	72.0 (1.4)	74.5 (5.9)	76.2 (4.4)	70.5 (4.3)	74.4 (4.2)	77.2 (5.9)

LI, low intensity exercise; HI, high intensity exercise. S_1_, S_2_ and S_3_, segment 1, segment 2 and segment 3.

## 4. Discussion

### 4.1. HRV Analysis

In this study, the components of heart rate variability (HRV) were affected strongly after exercise at 3800 m altitude (HA). Most values of total power and high frequency were significantly lower at 3800 m altitude than at sea level (Figure 2 and Figure 4). To best knowledge of the authors, no similar research has been conducted on this subject. Some researchers described an increase in sympathetic activity, or reduced vagal control of heart rate during acute exposure to hypoxia at resting [30,31]. Two authors in particular established that autonomic nerves activities were suppressed during exercise in hypoxia or ascent to altitude. These researchers have confirmed that parasympathetic activity decreased in hypoxia and/or exercise. In addition, the activities of CAM were related to the degree of hypoxia [32,33]. Our results demonstrate that the ratios of LF/HF during post-exercise at 3800 m altitude with 25% O_2_ and 0.5% CO_2_ (C1) and 3800 m altitude with 25% O_2_ and 3.0% CO_2_ (C2) (2600 m) were not statistically different from those found at SL, but were significantly different in HA. These findings have been supported by Kanai *et al.* who observed that the ratios of low frequency/high frequency at 2700 m and 3700 m were non-significant and significant, respectively, comparing with data at sea level [34]. The blunt responsiveness of cardiac autonomic modulation was considered a protection from excessive and long-term sympathetic stimulation during rest or exercise in hypoxia [35].

In this paper, the majority of low frequency/high frequency values (LF/HF) at 3800 m altitude with 25% O_2_ and 5.0% CO_2_ (C3) were notably lower than those at sea level (SL), but this significance did not appear in C1 and C2. In those conditions, CO_2_ concentration was the only factor that was different. In the C3 condition, the reduced LF/HF ratios with the inhalation of high concentration CO_2_ gas can be attributed to an increased high frequency component (HF) with no significant change in the low frequency component (LF) (Figure 3). These results were partially consistent with two studies that finding that high frequency power increased and low frequency/high frequency ratio decreased as CO_2_ was added to respiratory gas or a mild respiratory acidosis, which caused partial pressure of CO_2_ (${\text{P}}_{{\text{CO}}_{2}}$) in the blood to change [36,37]. No significant change in LF may suggest that ${\text{P}}_{{\text{CO}}_{2}}$ may not be influencing the baroreceptor. Data from the components of heart rate variability (HRV) in C1 and C2 conditions indicated that the cardiovascular system responded normally, even though the subjects undertook activities in these conditions and breathed this atmosphere for about 2 hours. These concentrations of inhaling CO_2_ gas would not elevate ${\text{P}}_{{\text{CO}}_{2}}$ enough to cause the change of HRV. However, it was difficult to explicitly distinguish the effect of HRV components using HRV analysis, and there was some doubt that very low frequency (VLF) and low frequency (LF) were the major determinants [29].

### 4.2. Heart Rate

Our results confirmed that maximum values of the heart rate and resting heart rate in HA were significantly higher than those at SL (Table 3). The changes of the heart rate to mild or moderate hypoxia are still under debate. Several studies have shown that heart rate in exercise or resting displayed little or no changes during acute exposure to mild hypoxia [34,38,39,40,41,42]. However, other studies have reported that maximal heart rate during maximal intensity exercise during acute exposure to mild hypoxia, changes significantly compared to the same measurements at sea level [43,44]. β-adrenergic sympathetic nerve stimulation and circulating catecholamines have been confirmed to be major factors contributing to the increase in heart rate that is observed during resting and submaximal exercise period at high altitudes [43,45,46]. When heart rate was not influenced by mild or moderate hypoxia, it might have been compensated by an enhanced extraction of oxygen by the muscle that was triggered by increasing circulating catecholamines [47].

In the present study, we showed that heart rate altered during exercise in C1, C2 and C3. Several researchers have obtained different results when subjects inhaled CO_2_ gas at different concentrations and for different durations. Generally, inhaling high concentrations of CO_2_ gas would induce increases in heart rate. However, Leihbold *et al.* reported that HR was unchanged and CO_2_ concentrations were not dose dependent, which was measured at 0%, 9%, 17.5% and 35% [24]. Sato and his colleagues confirmed that HR response to hypoxia did not display significant changes during exercise or CO_2_ inhalation [27]. Babb indicated that the maximal heart rate changed significantly during exhaustive exercise and inhalation of 3% CO_2_ [48]. Similar to the present study, Vercruyssen found that psychomotor and mentor performance displayed little change during submaximal exercise and inhalation of 3% or 4% CO_2_ in one hour, however, cardiovascular system parameters were not measured [49]. These authors concluded that the change in heart rate was relative to the level of ${\text{P}}_{{\text{CO}}_{2}}$ in the subject’s artery. In the present study, we showed that HR increased slightly during exercise in C1 and C2 and significantly in C3. These results indicate that heart rate was affected by inhalation duration and CO_2_ concentration (Table 3), both of which might raise the ${\text{P}}_{{\text{CO}}_{2}}$ level.

### 4.3. Blood Pressure

Several studies have demonstrated that SBP of normotensive subjects increased strongly with the increase in exercise intensity, while the DBP only changed slightly [50,51]. Our results show that SBP and DBP did not increase significantly during exercise and changed slightly during post-exercise in HA (Table 3 and Table 4). These findings are consistent with some previous studies which indicated that SBP and DBP experienced no change during exercise in hypoxia compared to normoxia [52,53]. Takamata *et al.* reported that compared to resting in hypoxia, the mean arterial pressure (MAP, calculated by SBP and DBP) was higher during exercise in hypoxia. The increase revealed a correlation with exercise intensity and was significant with exercise intensity of 45%–100% ${\dot{V}}_{O_{2\text{max}}}$ [54]. Ainslie confirmed that MAP increased non-significantly when the exercise intensity was 60%–70% ${\dot{V}}_{O_{2\text{max}}}$ [55]. Iwasaki pointed out that during resting in hypoxia, SBP, DBP and MAP changed indistinctively for the O_2_ inhalation of 19%, 17% and 15% compared to 21% O_2_ [56]. Blood pressure was reported to change slightly when subjects rested at an altitude of 5050 m [57]. Physical exercise led to an increase in cardiac output, and a rise in SBP was a natural consequence of dynamic exercise. Moreover, DBP changed slightly as a consequence of metabolic vasodilatation of the peripheral vessels [58]. As is known, cardiac output was determined by heart rate and stroke volume, the latter of which is a major factor for blood pressure change. In this study, increasing HR during exercise or resting in HA might satisfy the requirements of cardiac output. Accordingly, blood pressure increased slightly during exercise and resting in hypoxia.

Noting the combined effects of hypoxia, exercise and inhalation of CO_2_ gas, it was found that SBP increased significantly with exercise in C3, but non-significantly in C1 and C2. Furthermore, DBP varied slightly in C1, C2 and C3. Some previous studies indicated a change of blood pressure while inhaling high concentrations of CO_2_. For instance, inhaling 35% CO_2_ gas activated the hypothalamic-pituitary-adrenal (HPA) axis, which was followed by an increase in blood pressure [59,60,61]. In Luksch’s study, SBP and MAP significantly increased when subjects inhaled 92% O_2_ ± 8% CO_2_ for 10 min. However, while inhaling 95% O_2_ ± 5% CO_2_ and 97.5% O_2_ ± 2.5% CO_2_, no significant change was observed [62]. Schlibye showed that SBP and DBP values while exercising under the condition of hypoxia (4000 m) and 1.2% CO_2_ were similar to those while exercising in normoxia [63]. In present study, blood pressure increased in C3, which was probably attributed to a decreased vascular resistance which disappeared when subjects inhaled high concentration CO_2_ gas for too long, possibly leading to higher concentrations of CO_2_ in blood.

## 5. Conclusions

The present study investigated the changes of the cardiovascular system, as humans undertook activities in an oxygen enriched room with high concentrations of CO_2_ at high altitude. It was shown that as humans conducted physical activities in the oxygen-enriched room with 5.0% CO_2_ (3.14% at sea level) at 3800 m, the cardiovascular system demonstrated significant responses in terms of heart rate, blood pressure and cardiac autonomic regulation. However, there were no significant changes observed during exercise and breathing the air for about 2 h in the oxygen-enriched room with the CO_2_ concentrations of both 0.5% and 3.0% (0.31% and 1.89% at sea level) at 3800 m. That is, the difference in heart rate, blood pressure and HRV during exercise and breathing between the two conditions and sea level was not significant. Furthermore, whether or not the accumulated CO_2_ affects the cardiovascular system was determined by CO_2_ concentration, inhalation duration and the human body’s oxygen requirement. During exercise and resting in C1 and C2 conditions, inspired O_2_ can satisfy oxygen consumption of exercise and hypoxia. Under such environmental conditions, it was not found that CO_2_ concentration in the artery exceeded the normal physiological range. The results of our study preliminarily determined the level of CO_2_ that would had a significant influence on the cardiovascular system.

## 6. Research Limitations

Because exercise tests simulated a real exercise scenario in an oxygen-enriched room at high altitude, blood gas analysis was not performed on subjects. The present study did not measure and analyze the changes of ${\text{P}}_{{\text{CO}}_{2}}$ in the artery. On the basis of some previous studies, it was deduced that high CO_2_ inhalation would increase ${\text{P}}_{{\text{CO}}_{2}}$ level in the blood and further impact the cardiovascular system. Indeed, high concentration of CO_2_ was observed to have a significant impact on the cardiovascular system. The intervals of three CO_2_ concentration levels were large. In further study, the intervals of CO_2_ concentration levels will be shorten for ascertaining the concentration threshold of CO_2_ that will markedly affect the cardiovascular system.
